# An Endotoxin Tolerance Signature Predicts Sepsis and Organ Dysfunction at Initial Clinical Presentation

**DOI:** 10.1016/j.ebiom.2014.10.003

**Published:** 2014-10-07

**Authors:** Olga M. Pena, David G. Hancock, Ngan H. Lyle, Adam Linder, James A. Russell, Jianguo Xia, Christopher D. Fjell, John H. Boyd, Robert E.W. Hancock

**Affiliations:** a Centre for Microbial Diseases and Immunity Research, 2259 Lower Mall Research Station, University of British Columbia, Vancouver, British Columbia V6T 1Z4, Canada; b Flinders University Medical School, GPO Box 2100, Adelaide 5001, South Australia, Australia; c Division of Critical Care Medicine, University of British Columbia at St Paul's Hospital, P3311 1081 Burrard Street, Vancouver, British Columbia V6Z 1Y6, Canada; d Wellcome Trust Sanger Institute, Cambridgeshire, United Kingdom

**Keywords:** Sepsis, Severe sepsis, Diagnosis, Cellular reprogramming, Endotoxin tolerance, Signature, Immune dysfunction

## Abstract

**Background:**

Sepsis involves aberrant immune responses to infection, but the exact nature of this immune dysfunction remains poorly defined. Bacterial endotoxins like lipopolysaccharide (LPS) are potent inducers of inflammation, which has been associated with the pathophysiology of sepsis, but repeated exposure can also induce a suppressive effect known as endotoxin tolerance or cellular reprogramming. It has been proposed that endotoxin tolerance might be associated with the immunosuppressive state that was primarily observed during late-stage sepsis. However, this relationship remains poorly characterised. Here we clarify the underlying mechanisms and timing of immune dysfunction in sepsis.

**Methods:**

We defined a gene expression signature characteristic of endotoxin tolerance. Gene-set test approaches were used to correlate this signature with early sepsis, both newly and retrospectively analysing microarrays from 593 patients in 11 cohorts. Then we recruited a unique cohort of possible sepsis patients at first clinical presentation in an independent blinded controlled observational study to determine whether this signature was associated with the development of confirmed sepsis and organ dysfunction.

**Findings:**

All sepsis patients presented an expression profile strongly associated with the endotoxin tolerance signature (p < 0.01; AUC 96.1%). Importantly, this signature further differentiated between suspected sepsis patients who did, or did not, go on to develop confirmed sepsis, and predicted the development of organ dysfunction.

**Interpretation:**

Our data support an updated model of sepsis pathogenesis in which endotoxin tolerance-mediated immune dysfunction (cellular reprogramming) is present throughout the clinical course of disease and related to disease severity. Thus endotoxin tolerance might offer new insights guiding the development of new therapies and diagnostics for early sepsis.

## Highlights

• Addresses sepsis that affects up to 18 million persons annually with up to 5 million deaths worldwide.
• Describes an endotoxin tolerance gene expression signature associated with consecutive treatments with endotoxin and reflecting cellular reprogramming.
• Using a meta-analysis of the literature demonstrates the statistically significant association (p < 0.01; AUC 96.1%) of this endotoxin tolerance signature with early (day 1–3 after ICU admission) sepsis in 593 patients in 11 adult or child cohorts.
• Demonstrates, at first clinical presentation, the ability of this signature to predict the eventual diagnosis of sepsis and organ failure in an independent blinded controlled observational study of a unique cohort of possible sepsis patients.
• This study provides new insights that have the potential to guide the development of new therapies and diagnostics for early sepsis.

## 1 Introduction

Sepsis continues to be the major infection-related cause of death globally. In the United States alone, more than 120,000 persons die of sepsis each year (Martin et al., 2003). Despite modern medical advances including new antibiotics and vaccines, best practice treatments, and well-equipped intensive care units (Angus et al., 2001), sepsis mortality rates often remain high at ~ 30% (Lyle et al., 2014, Jawad et al., 2012). Bacterial endotoxins, such as lipopolysaccharide (LPS), are potent inducers of inflammation and have been suggested as triggers for the observed hyper-inflammation in sepsis, as well as the early life-threatening cytokine storm causing septic shock (Salomao et al., 2012). However despite these inflammatory components of sepsis, more than 30 clinical trials testing anti-inflammatory agents for the treatment of sepsis have shown no benefit (Lyle et al., 2014, Hotchkiss et al., 2013). This has contributed to a shift in our understanding of sepsis, from a condition of hyper-inflammation to one characterised by phases of inflammation and immune dysfunction/immunosuppression (Hotchkiss et al., 2013). Whilst our understanding of the immune dysfunction phase in sepsis remains limited (Lyle et al., 2014, Hotchkiss et al., 2013), one of the many hypotheses attempting to characterise the immune state in sepsis has suggested a role for endotoxin tolerance in the later stages of this process (Cavaillon et al., 2005, Otto et al., 2011, Schefold et al., 2008). Endotoxin tolerance, also termed cell reprogramming, can be defined as the severely reduced capacity of a cell to respond to LPS during a second exposure to this stimulus and represents an immune amnesia rather than an anti-inflammatory response (Cavaillon and Adib-Conquy, 2006). Other bacterial products can similarly induce reprogramming (Buckley et al., 2006). Despite some similarities in the cytokine production profiles of cells isolated from sepsis patients at late-stage sepsis time-points and in cells from in vitro endotoxin tolerance models (Cavaillon et al., 2005, Otto et al., 2011, Schefold et al., 2008), a robust link has yet to be made between sepsis and endotoxin tolerance. Obtaining a clear understanding of the inflammatory and immunosuppressive phases, including the clinical timepoints at which each occurs or predominates, is likely crucial to improving sepsis outcomes.

Here, we applied robust bioinformatics approaches to in-house and previously-published cohorts of early-stage sepsis patients. We showed that sepsis is characterised by an endotoxin tolerance phenotype that occurs very early during the clinical course of disease and is linked to disease severity. This places endotoxin tolerance as a novel therapeutic and diagnostic target in sepsis that may be used to predict the development of sepsis and organ failure in critically ill patients.

## 2 Materials and Methods

### 2.1 Gene Signature Definition and Analysis

**Fig. 1:**
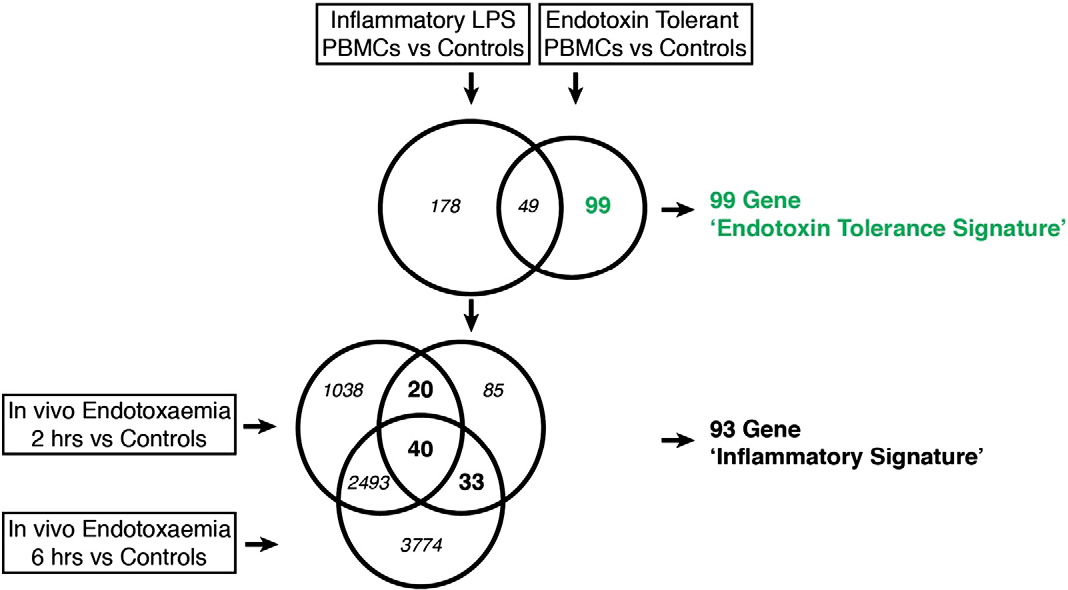
Definition of the ‘endotoxin tolerance signature’. Schematic representation of the method used to define the endotoxin tolerance signature and the inflammatory signature. The endotoxin tolerance signature was obtained from our previously published dataset (Pena et al., 2011) and defined as 99 genes uniquely differentially expressed in endotoxin-tolerant human PBMCs (treated twice with LPS), but not inflammatory human PBMCs (treated once with LPS), as compared to controls (fold change > 2, p-value < 0.05) (please see ref. Pena et al., 2011 for more details regarding this dataset). The inflammatory signature was reduced from the 178 genes uniquely differentially expressed in inflammatory human PBMCs to a common 93 gene signature, by selecting genes that were consistently differentially expressed in an in vivo human volunteer endotoxin challenge dataset (Calvano et al., 2005).

Endotoxin tolerance and inflammatory gene signatures were derived from our previously-published (Pena et al., 2011) microarray analyses of human PBMC identifying differentially-expressed genes compared to control PBMCs (GSE22248), with the inflammatory signature being delimited to genes that overlapped with a human volunteer endotoxin challenge (Calvano et al., 2005), as described in Fig. 1. Gene lists for the signatures are found in Supplementary Tables 1 and 2. Analysis of the presence or absence of the endotoxin tolerance and inflammatory signatures in patients and controls was performed using the well-established, statistically-rigorous gene set test ROAST (Wu et al., 2010), that asks whether a given set of genes/signature is enriched in a dataset. The ROAST method increases the strength of the test by additionally allowing the consideration of the direction of gene expression (Wu et al., 2010). The ROAST test is designed for any linear modelled data and was therefore suitable for both the microarray and RNA-Seq data, evaluated using the linear model in the Limma package (Wu et al., 2010, Smyth, 2004), ROAST, which delivers a specific p-value for the association of a given gene set with a particular condition (e.g. sepsis), was run with 99,999 rotations and so the lowest possible p-value arising from this test is 0.00001.

### 2.2 Meta-Analysis Datasets

A search without filter restrictions was performed in the US National Library of Medicine (PubMed) Database, the public repositories National Centre for Biotechnology Information-Gene Expression Omnibus (NCBI-GEO) and the European Bioinformatics Institute (EBI-ArrayExpress). The search terms used individually or in combination, include “sepsis”, “septic shock”, “septicaemia”, “bacteraemia”, “microarrays”, “RNA-Seq”, “cluster analysis”, “transcription profiling”, “gene expression”, “LPS”, “endotoxin”, “inflammation”, and “infection”. Datasets were also manually searched in review articles.

The final selection of datasets was based on the inclusion and exclusion criteria described in Supplementary Table 3. Within datasets, selected samples were excluded if they did not meet the inclusion criteria (e.g. SIRS patients that could not be classified as having sepsis due to lack of clinical information). In addition, exclusion criteria were: 1) studies only analysing a small number of genes (e.g.: RT-qPCR); 2) single-nucleotide polymorphism studies; 3) studies analysing only a single gene or pathway; 4) studies using resident immune cells (e.g. alveolar macrophages); and 5) studies using solid organ tissues.

It is important to mention that we found several datasets (especially with adult populations) using SIRS patients as controls. However, for this meta-analysis we selected datasets using healthy controls, as these allowed us to observe even minor transcriptional changes present in sepsis despite the stage of the disease (i.e. early or late stage). Parallel with this, we performed an initial in-house clinical study of 22 sepsis patients by analysing gene expression responses using RNA-Seq.

### 2.3 Patient Selection and New Clinical Study Design

In a blinded, observational, controlled cohort study, patients with suspected sepsis were identified when the attending physician activated the Institutional Severe Sepsis Order Set (Supplementary Fig. 1). Patients were enrolled from St. Paul's Hospital, Vancouver Canada, at the time of the first microbiological culture drawn for suspected sepsis. To determine the appropriate sample size for this study we used a standard power calculation for adequate sensitivity (Jones et al., 2003). To achieve a sensitivity of at least 0.9 at a 95% confidence level, we estimated a required sample size of 35 sepsis patients and 70 patients total (assuming that 50% of patients with a suspicion of sepsis actually have sepsis). We recruited 72 total patients which proved subsequently to include 37 sepsis patients. The inclusion criteria for this study were the suspicion of sepsis by the attending physician, activation of the Institutional Severe Sepsis Order Set, and successfully obtaining samples for microbial cultures. The majority of patients (83%) were enrolled from the emergency room. As shown in Supplementary Table 4, these individuals were heterogeneous. Our UBC ethical approval protocol enabled deferred consent allowing early patient recruitment in cohorts that spanned from non-infected to septic shock. As controls, we recruited consented healthy individuals, with no evidence of infection, who were scheduled for non-urgent surgery. Blood was collected in EDTA tubes at the time of initial blood culture, and immediately placed on ice. Plasma and buffy coat were separated and two 1-ml aliquots transferred into bar-coded cryovials at − 20 °C until they were transferred to a secure, alarmed − 80 °C freezer. Study identification numbers were assigned to the secured enrolment forms and used during all subsequent analyses; thus researchers analysing gene expression in these patients were blinded as to patient identity or clinical course, which was only revealed during final data analysis. Clinical data was stored in an ORACLE-based database on a firewalled, RSS encrypted server at St Paul's Hospital.

Clinical data was collected retrospectively by physician researchers blinded to the RNA-Seq data. Sepsis was retrospectively defined as suspected or proven infection in addition to at least two of the following assessments: Initial WBC, < 4000 or > 12,000 per μl; Triage Temperature < 36C or > 38 °C; and Triage Heart Rate, > 90 bpm. New organ dysfunction was defined as outlined in Supplementary Table 5 and based on laboratory values collected in the electronic medical record system. Initial vital signs were retrospectively extracted from the paper records.

### 2.4 Ethical Conduct of Research

All studies were performed under UBC ethics approval [IDs H11-00505 for patient sample collection and H08-00293 for RNA-Seq analysis]. Our UBC ethical approval protocol H11-00505 enabled deferred consent allowing early patient recruitment in cohorts that spanned from non-infected to septic shock and subsequent approval by patients.

### 2.5 RNA-Seq

Transcriptomic analysis was performed by the high throughput sequencing of cDNAs (RNA-Seq). cDNA libraries were prepared from total RNA using the TruSeq Stranded Total RNA Sample Prep Kit with a Ribo-Zero sample preparation guide (Illumina). RNA-Seq was performed on a GAIIx instrument (Illumina), using a single read run of 63 bp-long sequence reads (+ adapter/index sequences). A standard analysis protocol was used whereby raw basecall data was converted to FASTQ sequence files using Off-Line Basecaller 1.9.4 (Illumina) and a custom Perl script. Reads were aligned to the hg19 human genome with TopHat version 2.06 and Bowtie2 2.0.0-beta6 (Trapnell et al., 2009) and mapped to Ensembl transcripts. Raw data was deposited into NCBI GEO.

### 2.6 Data Analysis

All data processing was performed in R using Bioconductor modules (Gentleman et al., 2004). For the meta-analysis, normalised datasets were downloaded from NCBI GEO using the Bioconductor package GEOquery (Davis and Meltzer, 2007). An additional quantile normalisation step was included if the data required further normalisation. For the RNA-Seq analysis, data was normalised using the Voom function in the Limma package which converts read counts to weighted log base 2 counts per million. For both the meta-analysis and RNA-Seq analyses, data was summarised using the linear model in the Limma package (Smyth, 2004, Gentleman et al., 2004).

### 2.7 Classification Analysis

Each dataset was split into training (containing 2/3 of sepsis patients and controls) and test (containing 1/3 of sepsis patients and controls) sets, using random sampling. A model was defined on the training set and then assessed on the test set using the randomForest package (Liaw and Wiener, 2002) with ntree set to 1000. The procedure was repeated 100 times, and the average AUC values were recorded for each dataset. Note that AUC (also known as AUROC) = Area Under Receiver Operator Curve (TPR (Sensitivity) vs. FPR (1-Specificity) curve) is an indicator of the accuracy of diagnosis, based on the Endotoxin Tolerance Signature, for the compared groups. AUC values were calculated using the ROCR package (Sing et al., 2005).

## 3 Results

### 3.1 Endotoxin Tolerance Signature in Early Sepsis

To assess whether endotoxin tolerance (cellular reprogramming) contributes to the immune dysfunction observed in sepsis, we first defined genetic signatures of endotoxin tolerance and inflammation (Fig. 1). The ‘endotoxin tolerance signature’ (Supplementary Table 1) comprised 99 genes that were uniquely differentially expressed in endotoxin-tolerant PBMCs, but not inflammatory PBMCs, as compared to non-LPS-exposed controls (Pena et al., 2011). An ‘inflammatory signature’ (Supplementary Table 2) was defined based on genes differentially regulated in inflammatory PBMCs but not in endotoxin-tolerant PBMCs by combining dysregulated genes present in our published dataset (Pena et al., 2011) and an experimental human endotoxin-challenge dataset (Calvano et al., 2005).

We first recruited an initial cohort of 22 adult patients with confirmed sepsis at various timepoints (1 to 3 days after the initial clinical suspicion of sepsis) throughout the early clinical course of disease. To increase the number of sepsis patients in this analysis and to reduce the limitations of a single-centre study, we also performed a retrospective global meta-analysis on 10 published, independent and blinded clinical sepsis cohorts, encompassing 571 early sepsis patients (1 or 3 days post-ICU admission) and 160 healthy controls (Supplementary Table 3). Healthy controls were used as the basis for comparison to allow for the detection of smaller changes in gene expression and to limit study–study variability in the control population used as baseline.

**Fig. 2:**
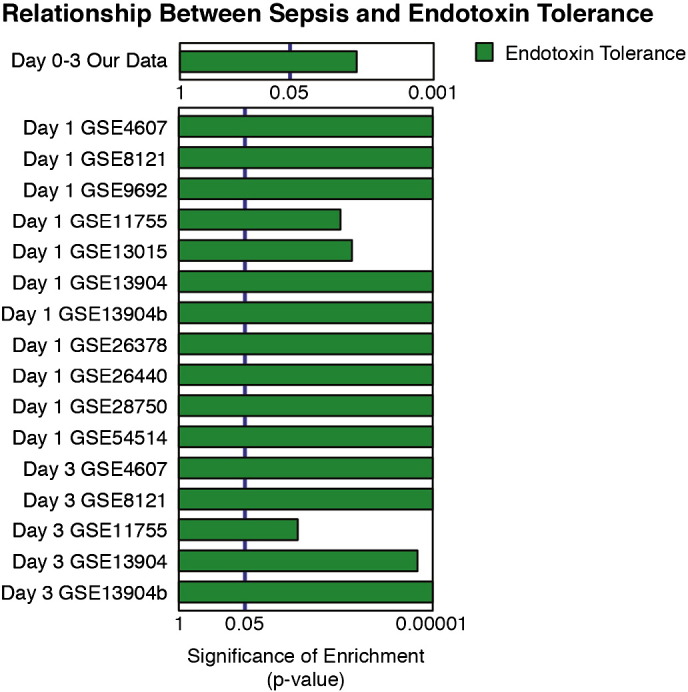
Sepsis patients from published datasets showed a strong association with the ‘endotoxin tolerance signature’. A gene-set test approach, ROAST (Wu et al., 2010), testing the statistically significant presence of a signature (collection) of genes, was used to characterise the enrichment of ‘Endotoxin Tolerance’ in sepsis patients versus controls from a small study performed by us and 10 previously published datasets. All datasets contained sepsis patients recruited at day 1 or 3 post-ICU admission and were compared to ‘healthy’ controls. The ROAST gene-set test was run with 99,999 rotations so the most significant p-value resulting from this test is 0.00001. p-Values from the ROAST gene-set test were graphed as log (1/p-value), and untransformed p-values are shown for ease of visualization.

To assess the relative expression of the Endotoxin Tolerance and inflammatory signatures in sepsis patients versus healthy controls, we used a gene-set test approach, which examines whether there is differential enrichment of a given signature (gene-set) between groups (Wu et al., 2010). We found that sepsis patients in all 11 cohorts (in-house and meta-analysis) showed an immunological expression profile strongly associated with the endotoxin tolerance signature when compared to controls (Fig. 2). Whilst the inflammatory signature was significantly associated with eight of the datasets, this association was consistently weaker than for the endotoxin tolerance signature (Supplementary Fig. 2). In contrast to previous reports associating endotoxin tolerance only with late stage sepsis (Cavaillon et al., 2005, Otto et al., 2011, Schefold et al., 2008), the association with the ‘endotoxin tolerance signature’ was present in sepsis patients as early as Day 1 post-ICU admission, and was maintained on Day 3, consistent with the early development of a ‘stable’ endotoxin tolerance profile in sepsis patients (Fig. 2). Thus the early immune dysfunction in Sepsis appeared to be characterised by endotoxin tolerance/cellular reprogramming.

### 3.2 Endotoxin Tolerance Signature at First Clinical Presentation

We next sought to better understand the timing of endotoxin tolerance development in early sepsis. To do this, we recruited a unique blinded, prospective, observational cohort of patients at the earliest possible stage of clinical disease. Patients were recruited immediately after clinical suspicion of sepsis (i.e. prior to diagnosis), based on the attending physician's physical examination and request for microbial culture testing. RNA-Seq was performed on RNA isolated from the initial blood sample taken for cultures to aid in sepsis diagnosis/microbial identification. We recruited 72 very early suspected sepsis patients (sufficient power to achieve 90% sensitivity, see the Materials and Methods), as well as 11 control patients recruited prior to elective surgery with no underlying morbidities (Supplementary Table 4). Since only a subset of the patients in this ‘critically ill’ patient cohort would go on to develop true sepsis, this cohort represented a clinically-challenging cohort of patients who initially presented with variable serious derangements in physiology (potentially caused by sepsis).

**Fig. 3:**
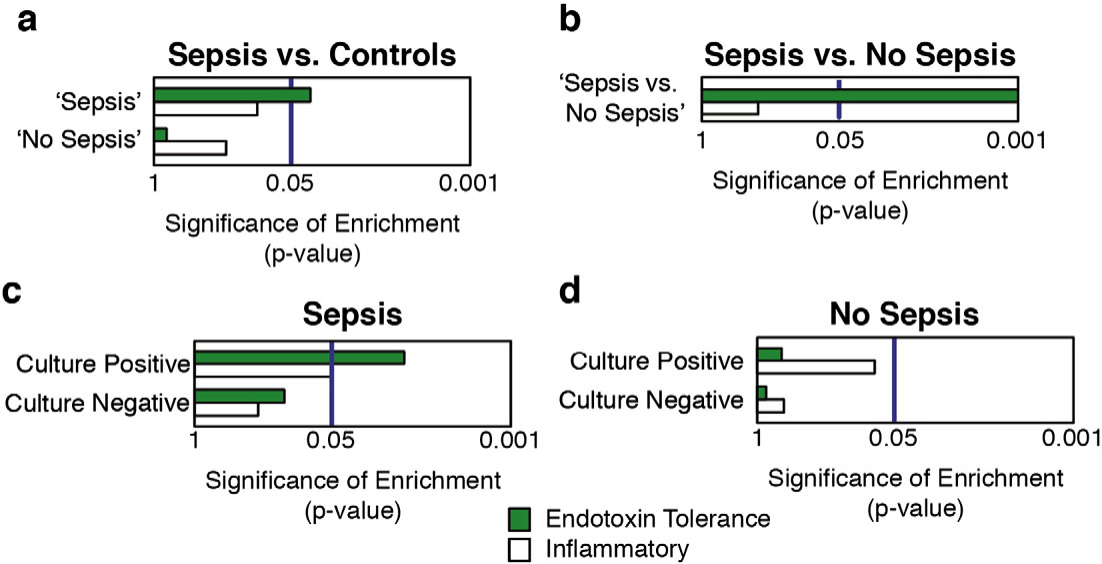
The ‘endotoxin tolerance signature’ was strongly associated with sepsis patients at first clinical presentation. A gene-set test approach (Wu et al., 2010) was used to characterise the enrichment, cf. controls as well as non-sepsis critically ill patients, of the ‘Endotoxin Tolerance’ and ‘Inflammatory’ signatures in prospective sepsis patients from a unique in-house cohort recruited on first clinical suspicion of sepsis (i.e. generally in the emergency ward and before ICU admission cf. the studies described in Fig. 2 that were post-ICU admission). Patient groups were subsequently defined based on retrospective clinical characteristics as ‘Sepsis’ or ‘No Sepsis’ consistent with the current sepsis criteria^3,15,16,36^ (Supplemental Table 4). Analyses were performed comparing (a) the ‘Sepsis’ and ‘No Sepsis’ groups vs. controls and (b) the ‘Sepsis’ and the ‘No Sepsis’ groups to each other. Additionally, enrichment of the signature was also analysed based on microbial culture results within (c) the ‘Sepsis’ group and (d) the ‘No Sepsis’ group.

Based on secondary clinical assessments following sample isolation (Supplementary Table 4), patients were retrospectively classified as ‘Sepsis’ (n = 37), or ‘No Sepsis’ (n = 35), consistent with current sepsis diagnostic criteria (see the Materials and Methods) (Lyle et al., 2014, Bone et al., 1992, Vincent et al., 2013, Levy et al., 2003). Strikingly, even at the earliest stage of clinical sepsis, the endotoxin tolerance signature was significantly enriched in patients who were subsequently confirmed to have sepsis (‘Sepsis’ group), but not in those with other diagnoses (‘No Sepsis’ group) (Fig. 3a). Whilst the inflammatory signature did not reach statistical significance in the ‘No Sepsis’ group, the contrasting relative enrichment of the Endotoxin Tolerance and inflammatory signatures in the 2 groups may indicate a fundamental difference in the balance of endotoxin tolerance and inflammation unique to sepsis patients (Fig. 3a).

The endotoxin tolerance signature was also enriched in the ‘Sepsis’ group when directly compared to the ‘No Sepsis’ group (Fig. 3b), which supports the specificity of endotoxin tolerance to sepsis and not just to ‘ill’ patients. To further exclude the possibility that the signature was only detecting critically ill patients, we also determined that the endotoxin tolerance signature was not significantly enriched in acute kidney transplant rejection (p value = 0.213) or myocardial infarction (p value = 0.433) patients (Kurian et al., 2014, Silbiger et al., 2013), compared to healthy controls. Together these data suggest that endotoxin tolerance is present throughout the initial clinical course of sepsis, detectable before ‘diagnosis’, and can be used to differentiate patients who develop sepsis in a cohort of patients where there was a suspicion of sepsis.

Current definitions of sepsis (Lyle et al., 2014, Bone et al., 1992, Vincent et al., 2013, Levy et al., 2003) refer to suspected or confirmed infection together with other systemic abnormalities such as initial WBC count, triage temperature and heart rate, whilst severe sepsis usually involves one or more organ failures. All patients here were suspected to have infection upon first clinical presentation which was confirmed in 19 of the 37 individuals who were eventually diagnosed with sepsis (Supplementary Table 4). Nevertheless, similar levels of significance of association of the endotoxin tolerance signature with sepsis were observed for this group as a whole, and for that subset of the group with confirmed infections (p < 0.01). To further highlight this concept, we compared signature enrichment in the ‘Sepsis’ and ‘No Sepsis’ groups following further separation based on microbial culture results. Separating the groups based on culture results did not change the overall associations between endotoxin tolerance and the ‘No Sepsis’ group (Fig. 3d). Indeed amongst the ‘Sepsis’ group, the endotoxin tolerance signature was significantly enriched in the culture positive group, although there was a trend towards enrichment in the culture negative group (Fig. 3c). This is consistent with the sensitivity issues of bacterial culturing methods and the challenge in diagnosing true infection-positive patients in those with suspected infection. Moreover, since our RNA-Seq analysis was performed on the same blood samples used for diagnostic microbial cultures, the strong association between sepsis and our endotoxin tolerance signature suggests that this signature might provide a more sensitive tool for diagnosis than microbial culture.

**Fig. 4:**
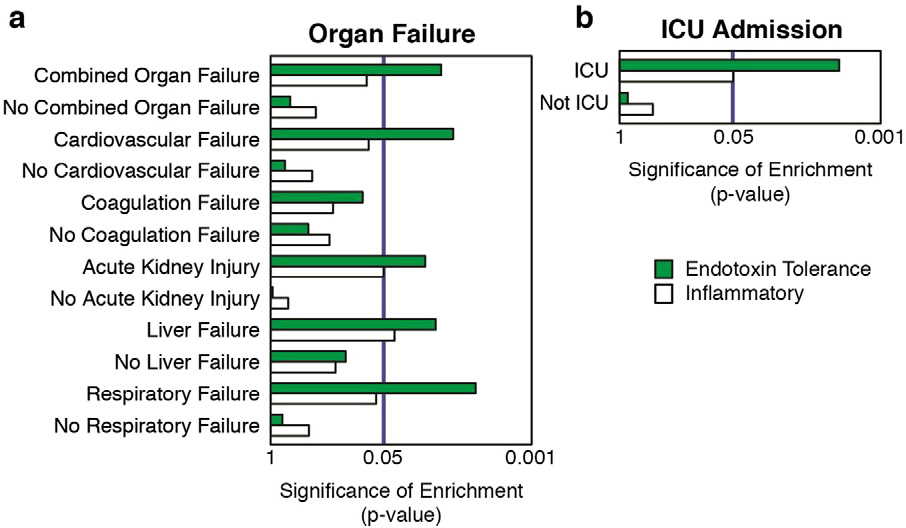
The ‘endotoxin tolerance signature’ was strongly associated with sepsis patients at first clinical presentation and was associated with the severity of the disease. A gene-set test approach (Wu et al., 2010) was used to characterise the enrichment, cf. surgical controls, of the ‘Endotoxin Tolerance’ and ‘Inflammatory’ signatures in prospective sepsis patients from a unique in-house cohort recruited on first clinical suspicion of sepsis (i.e. generally in the emergency ward prior to ICU admission). (a) Patients were grouped into individual-, combined- (3 +), individual type of organ failure and no-organ failure groups. NB. No patients were observed with sepsis-associated encephalopathy. (b) Patients were also grouped into those requiring and those not-requiring transfer to the ICU.

It has been commonly postulated that the immunosuppressive state in sepsis is related to the end-stage development of organ dysfunction, and so we sought to address this using our clinical cohort. Subsequent organ dysfunction development (cardiovascular, coagulation, kidney, liver, and respiratory, Supplementary Tables 4, 5) was assessed up to 48 h after study enrolment, with patients retrospectively grouped into organ-dysfunction positive and negative groups, independent of sepsis diagnosis. These groups were then subjected to the same gene-set test analysis, as above. Interestingly, the endotoxin tolerance signature was found to be significantly associated with the development of several individual and multiple (3 +) organ dysfunction(s) (Fig. 4a). Although ICU admission may depend on the inherent subjectivity of hospital practice, such as space or number of beds available in each department, patients that are moved to the ICU are generally in a deteriorating condition with an increased risk of mortality. Therefore, we also assessed the requirement for ICU admission as a second, less precise measure of disease severity and showed that endotoxin tolerance signature was again associated with this indicator of increased disease severity (Fig. 4b). These results indicated that endotoxin tolerance appears to be associated with sepsis severity and specifically with the downstream development of organ failure.

### 3.3 Prognostic Potential

**Fig. 5:**
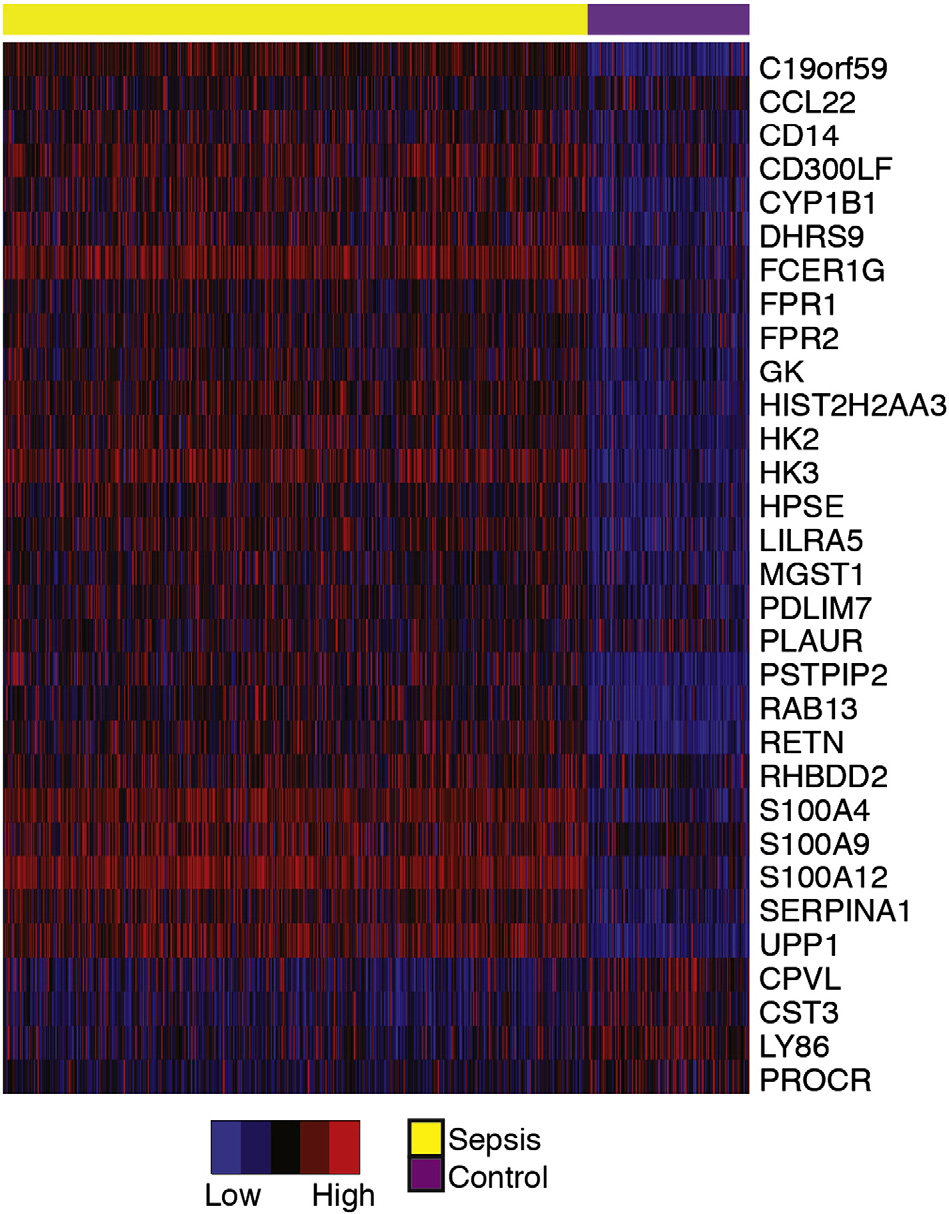
A core-set of endotoxin tolerance genes characteristic of sepsis patients. A core-set of 31 of the 99 genes from the ‘endotoxin tolerance signature’ was determined based on the most frequently differentially expressed genes observed literature sepsis datasets. For better visual comparison across different studies, each individual dataset was further transformed by dividing gene expression values into six equal bins. Data is presented as a heatmap with blue and red representing relatively low and high expression, respectively.

Given the strong association between endotoxin tolerance and sepsis across more than 600 patients from 11 independent datasets, we hypothesised that our signature would be a useful tool in sepsis diagnosis. Whilst the full 99 gene, endotoxin tolerance signature was useful for characterising the immune dysfunction in sepsis, a smaller number of genes would be of more use in a diagnostic test. Thus we first sought to reduce our 99-gene signature to its essential component, prior to testing its diagnostic utility. To do this, we selected genes that showed greater than 1.5 fold differential expression between sepsis patients and controls across the majority (7 +) of the 10 literature datasets. This identified a core-set of 31 genes from the original 99 gene endotoxin tolerance signature (Fig. 5). It is worth noting that many of the genes in our endotoxin tolerance signature are individually dysregulated in other disease states; however the combination appeared specific for sepsis.

We then used the classification algorithm randomForest to preliminarily assess the diagnostic utility of our 31 gene core-set to classify sepsis patients. We divided each dataset (external and internal) into training and test sets and performed randomForest classification independently on each dataset. The core-set showed excellent performance when separating sepsis patients from controls across all datasets (average AUC of 96.1%), and when separating patients who subsequently developed sepsis or individual/combined organ failure in our cohort of patients with a suspicion of sepsis (AUCs from 74.1 to 84.7%, with liver failure lower at 54.1%) (Table 1). The core-set showed improved performance when classifying Sepsis patients with confirmed infection (70.4%), requiring ICU admission (73.6%), and with at least 1 organ failure (75.2%) suggesting that our endotoxin tolerance signature shows increased diagnostic performance (and clinical relevance) when identifying patients who go on to develop definitive sepsis and more serious disease (Table 1). Similar results were obtained with the full 99 gene endotoxin tolerance signature (Table 1). The strong performance of our classifier across multiple distinct datasets and at a clinically relevant timepoint (blood draw for diagnostic cultures) supports the potential use of our classifier in the diagnosis of sepsis.

**Table 1:** Diagnostic potential of the endotoxin tolerance signature. Each dataset was split into training (containing 2/3 of sepsis patients and controls) and test (containing 1/3 of sepsis patients and controls) sets using random sampling. Datasets GSE13015 and GSE11755 were omitted from this analysis due to low numbers of controls (N = 3) in each dataset. For each of the remaining 8 datasets, the model was defined on the training set and then assessed on the test set using the randomForest package (Xiu and Jeschke, 2013) with ntree set to 1000. The procedure was repeated 1000 times, and the average AUC (Area Under Receiver Operator Curve) values (representing the accuracy of the diagnosis), recorded for each dataset. This analysis was repeated on our dataset to classify patients with an initial suspicion of sepsis who did or did not go on to develop sepsis or organ failure.

Variable	AUC using 31 gene endotoxin tolerance core-set	AUC using 99 gene endotoxin tolerance signature
*Sepsis (patient numbers in brackets) vs. controls*		
In-house Sepsis study #1 (22) vs. controls	78.4%	77.8%
In-house Sepsis study #2 (37) vs. controls	98.1%	97.1%
GSE28750 study (30) vs. controls	100%	100%
GSE9692 study (45) vs. controls	99.4%	99.3%
GSE13904 study (227) vs. controls	97.8%	97.9%
GSE26440 study (130) vs. controls	99.2%	99.1%
GSE4607 study (84) vs. controls	98.4%	98.3%
GSE8121 study (71) vs. controls	98.1%	98.1%
GSE26378 study (70) vs. controls	100%	100%
GSE54514 study (35) vs. controls	91.5%	94.4%
Mean	96.1%	95.9%

*Sepsis vs. No Sepsis — study #2*		
Sepsis vs. No Sepsis	63.9%	66.4%
Sepsis (Positive Culture vs. No Sepsis)	70.4%	75.7%
Sepsis (ICU vs. No Sepsis)	73.6%	75.6%
Sepsis (1 + Organ Failure vs. No Sepsis)	75.2%	74.5%

*Organ Failure vs. No Organ Failure — study #2*		
Respiratory	84.7%	84.8%
Cardiovascular	84.1%	82.0%
Liver	54.1%	55.3%
Acute kidney injury	76.7%	79.4%
Coagulation	74.4%	77.3%
Combined (3 +)	78.4%	77.6%

## 4 Discussion

The association between the endotoxin tolerance signature and confirmed sepsis was strong and statistically significant in a total of 12 distinct datasets (Fig. 2, Fig. 3) and as such was independent of sample size, location, method, gender, age and ethnicity. These results are consistent with our hypothesis that the endotoxin tolerance signature is robustly associated with very early sepsis. The endotoxin tolerance signature was also associated with disease severity measured primarily by the development of organ dysfunction. Therefore, we propose here an updated model of sepsis pathogenesis mediated by an endotoxin tolerance-mediated immune dysfunction. This is consistent with but further clarifies a recent study (Hotchkiss et al., 2013) that suggested that early sepsis was associated with coincident inflammatory and anti-inflammatory/immunosuppressive responses. It is worth mentioning that endotoxin tolerance is not an anti-inflammatory state per se but rather a cellular reprogramming (which also occurs with Gram positive bacteria) that leads to immune amnesia, disabling responses to agonists like endotoxin (Cavaillon and Adib-Conquy, 2006, Buckley et al., 2006, Pena et al., 2011). We also demonstrated that this immune dysfunction could be detected at a clinically relevant ‘diagnostic’ time-point, providing unique information regarding the patients' functional immune status. In the future, our genetic classifier could help to define a subset of patients who might benefit from immunomodulation (e.g. anti-endotoxin tolerance) and supportive therapies.

Sepsis has been traditionally classified as an early stage excessive inflammatory state followed by a transition to a late stage anti-inflammatory/immunosuppressive state (e.g. endotoxin tolerance) (Cavaillon et al., 2005, Otto et al., 2011, Schefold et al., 2008). However, a model of concurrent immunosuppression and hyperinflammation has been recently hypothesised to explain the complex pathogenesis of sepsis at various timepoints (Hotchkiss et al., 2013). The current study indicates that immunosuppression is being driven by endotoxin tolerance that occurs at a much earlier stage of clinical disease than previously appreciated, consistent with the failure of immunosuppressive treatments in more than 30 clinical trials (Lyle et al., 2014). In agreement with the concurrent inflammation/immunosuppression model, we observed significant enrichment of both the endotoxin tolerance and inflammatory signatures at all early-stages of disease, although the endotoxin tolerance signature dominated. If there is an immunological phase characterised solely by excessive inflammation, our data would suggest that this occurs pre-clinically (Supplementary Fig. 3). Although ‘pre-clinical’ disease can represent a relatively large time frame, it is less clinically relevant from a therapeutic/intervention perspective. One possible explanation for the concurrent occurrence of both inflammation and immunosuppression may lie at the immune cell population level. Due to their continuous replenishment from the bone marrow (Summers et al., 2010), neutrophils, which show very little gene expression, are potential drivers of pro-inflammatory cytokine responses, even as longer-lived monocyte/macrophage populations and other perhaps antigen presenting cells (dendritic cells and B lymphocytes) are being locked into and driving the endotoxin tolerance/cellular reprogramming response (Parnell et al., 2013). Therefore, at a systemic level, sepsis appears to be typified by a combination of neutrophilic inflammation, driving vascular leakage, coagulation, lymphocyte death, etc. (Hotchkiss and Karl, 2003, de Jong et al., 2010), and monocytic/macrophage reprogramming, impairing immune responses to primary and secondary infections (Hotchkiss et al., 2013, Leentjens et al., 2013, Xiu and Jeschke, 2013). Future studies aimed at assessing the development of cellular reprogramming over time (instead of a single timepoint) and at a cellular level will help clarify these processes. In addition, it will be interesting to examine patients with severe trauma (Hotchkiss et al., 2013) to see if they also have the endotoxin tolerance signature, despite the possibility that they are non-infectious. However, performing longitudinal studies on suspected sepsis patients recruited with deferred consent is logistically challenging.

The other important observation in relation to the balance between inflammation and endotoxin tolerance, is the relative enrichment of each signature in critically ill patients who do or do not develop sepsis (Fig. 3). From a biological perspective, our observations may suggest that in individuals with localized infections (e.g. patients in the No Sepsis group), when an initial insult occurs, the brief inflammatory response quickly subsides to balance inflammation and bring the system to homeostasis. However, in sepsis, where there is an uncontrolled source of infection, and possible contributing genetic factors (Murkin and Walley, 2009), the immunological balance between inflammation and endotoxin tolerance becomes detrimentally unbalanced towards a state increasingly dominated by endotoxin tolerance (Supplementary Fig. 3). This model is supported by our observed association between the endotoxin tolerance signature and disease severity/organ dysfunction (Fig. 4). Organ dysfunction is considered the main factor contributing to patient deterioration and ultimately death. Importantly, the endotoxin tolerance signature was present up to 48 h prior to the development of organ dysfunction, suggesting that this signature might be additionally used as a screening method to assess which patients are at a higher risk for developing a worsening condition. Moreover, network analysis (Xia et al., 2014) of the Endotoxin Tolerance genes revealed that 53 of the genes formed a very tight protein–protein-interaction sub-network suggesting that the signature may identify key genes related to immune dysfunction (and possibly susceptibility to infection) in sepsis patients (Supplementary Fig. 4).

Whilst the current study was primarily focused on classifying the immune dysfunction in sepsis, the extremely strong association between endotoxin tolerance and sepsis leads us to explore the potential use of our signature in diagnosis. Indeed, both the gene-set and classification analyses support this use (Table 1, Fig. 1, Fig. 2, Fig. 3). However, one limitation of this analysis was the relatively low patient numbers in some of the datasets (including our in-house datasets). Whilst the ROAST gene-set test (Wu et al., 2010) was designed for much lower group numbers, classification tests (such as randomForest (Xiu and Jeschke, 2013)) are optimally used with larger patient numbers. Thus larger cohort studies, specifically designed for assessing the potential of this signature as a diagnostic tool (especially for the prediction of organ failure in sepsis patients), will likely be required to confirm its potential as a diagnostic signature. However, as the association of endotoxin tolerance and sepsis was robustly identified in 12 distinct datasets, endotoxin tolerance is likely to be clinically relevant (as a potential key factor in sepsis aetiology and/or therapeutic target), regardless of the diagnostic utility of our specific signatures. In this regard there are a number of agents in development for unlocking an M2 macrophage state (Sica and Mantovani, 2012) that drives endotoxin tolerance (Pena et al., 2011).

In conclusion, we have provided a description of a unique endotoxin tolerance gene expression profile, present very early in the course of sepsis, and linked to sepsis pathogenesis and the risk of developing organ dysfunction. The results of this study should be further tested prospectively in a large multicenter cohort of patients with sepsis using current definitions for infection, sepsis, severe sepsis, and multiple system organ failure.

## Author Contributions

OMP was involved in defining the initial concept and designing the study, discovering the endotoxin tolerance signature, and writing the manuscript; DGH performed most of the final bioinformatics and participated in writing the manuscript; NHL was involved in retrieving the clinical data from the medical records and editing the manuscript; AL was involved in retrieving the clinical data from the medical records and critiquing the manuscript; JAR was involved in discussing and critiquing the findings throughout the study and editing the manuscript; JX was involved in initial bioinformatic analyses, especially network analyses, and provided essential advice regarding determining diagnostic accuracy; CDF was involved in bioinformatic analyses including analysis of raw data from RNA-Seq, and downloading of relevant studies from GEO; JHB was involved in discussing the initial concepts and conducting the in-house clinical study as well as editing the manuscript; and REWH was involved in defining the initial concept and design of the study, supervising all aspects of the research, and in extensively editing the manuscript.

## Role of the Funding Source

The funding source, 10.13039/501100002879Canadian Institutes of Health Research (MOP-74493 and MCT-44152), provided support for this research but was not involved in any aspect pertinent to the study.

## Competing Interests

The authors declare no relevant conflicts of interest. The study depicted here has been filed for a US provisional patent application 61/953,458 (inventors Robert Hancock, Olga Pena, David Hancock and John Boyd) which claims the use of the signature for diagnosing sepsis and the use of molecules that reverse the endotoxin tolerant state of immune cells as a therapeutic strategy.

## Appendix A Supplementary Data

An endotoxin tolerance signature predicts sepsis and organ dysfunction at first clinical presentation.
