# Supplementary material for: An Endotoxin Tolerance Signature Predicts Sepsis and Organ Dysfunction at Initial Clinical Presentation
Source: eBioMedicine. 2014 Oct 7;1(1):64–71. doi: 10.1016/j.ebiom.2014.10.003 (PMC4326653; doi:10.1016/j.ebiom.2014.10.003)
Supplement: Supplementary file 1 — An endotoxin tolerance signature predicts sepsis and organ dysfunction at first clinical presentation. [file mmc1.pdf]

SUPPLEMENTARY INFORMATION

AN ENDOTOXIN TOLERANCE SIGNATURE PREDICTS SEPSIS AND ORGAN DYSFUNCTION AT INITIAL CLINICAL PRESENTATION

Olga M Pena, David G Hancock, Ngan H Lyle, Adam Linder, James A. Russell, Jianguo Xia, Christopher D. Fjell, John H. Boyd, and Robert E W Hancock

SUPPLEMENTARY FIGURES

Supplementary Figure 1: Institutional Severe Sepsis Order Set for management of severe sepsis and septic shock at St Paul's Hospital where our studies were performed.

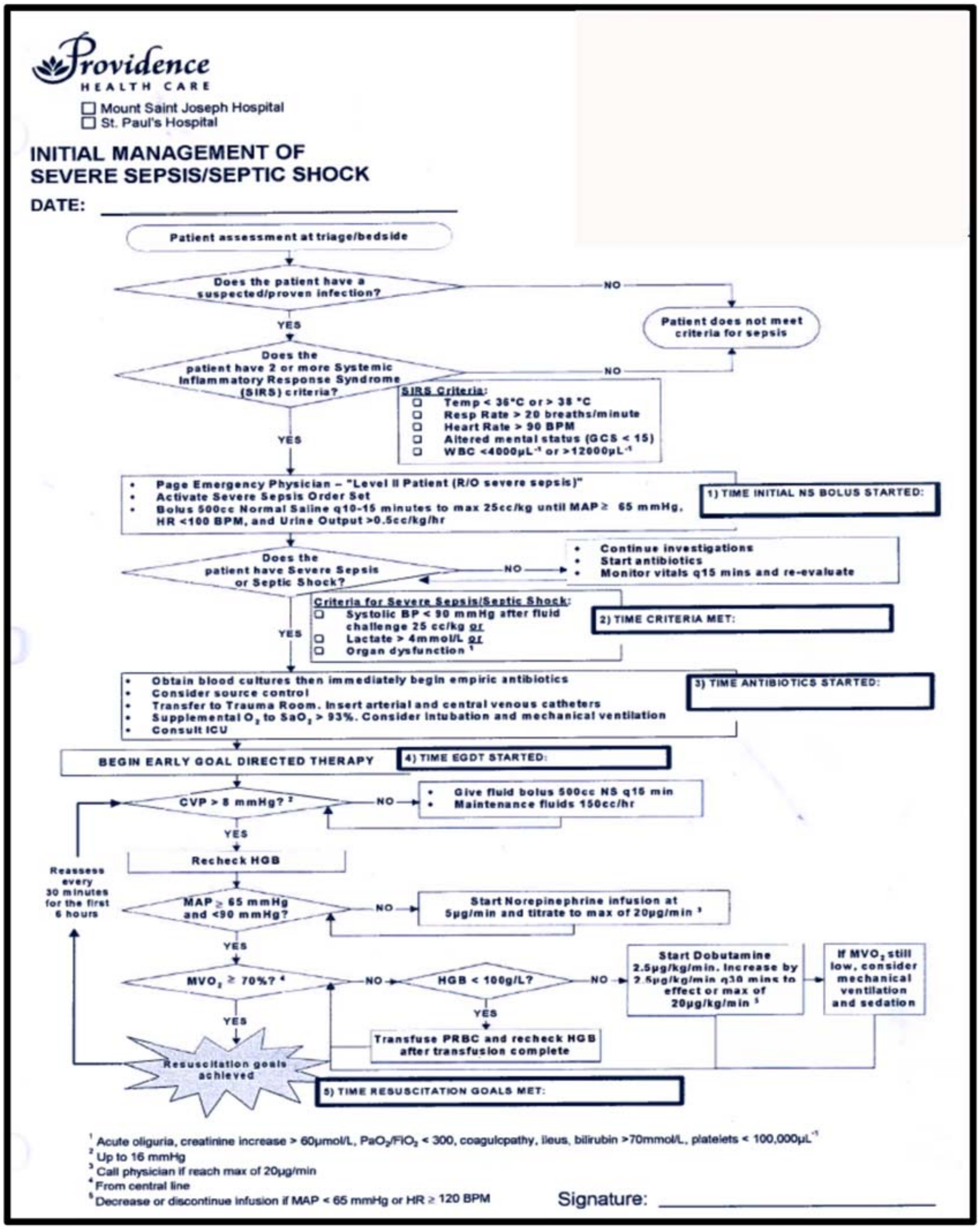

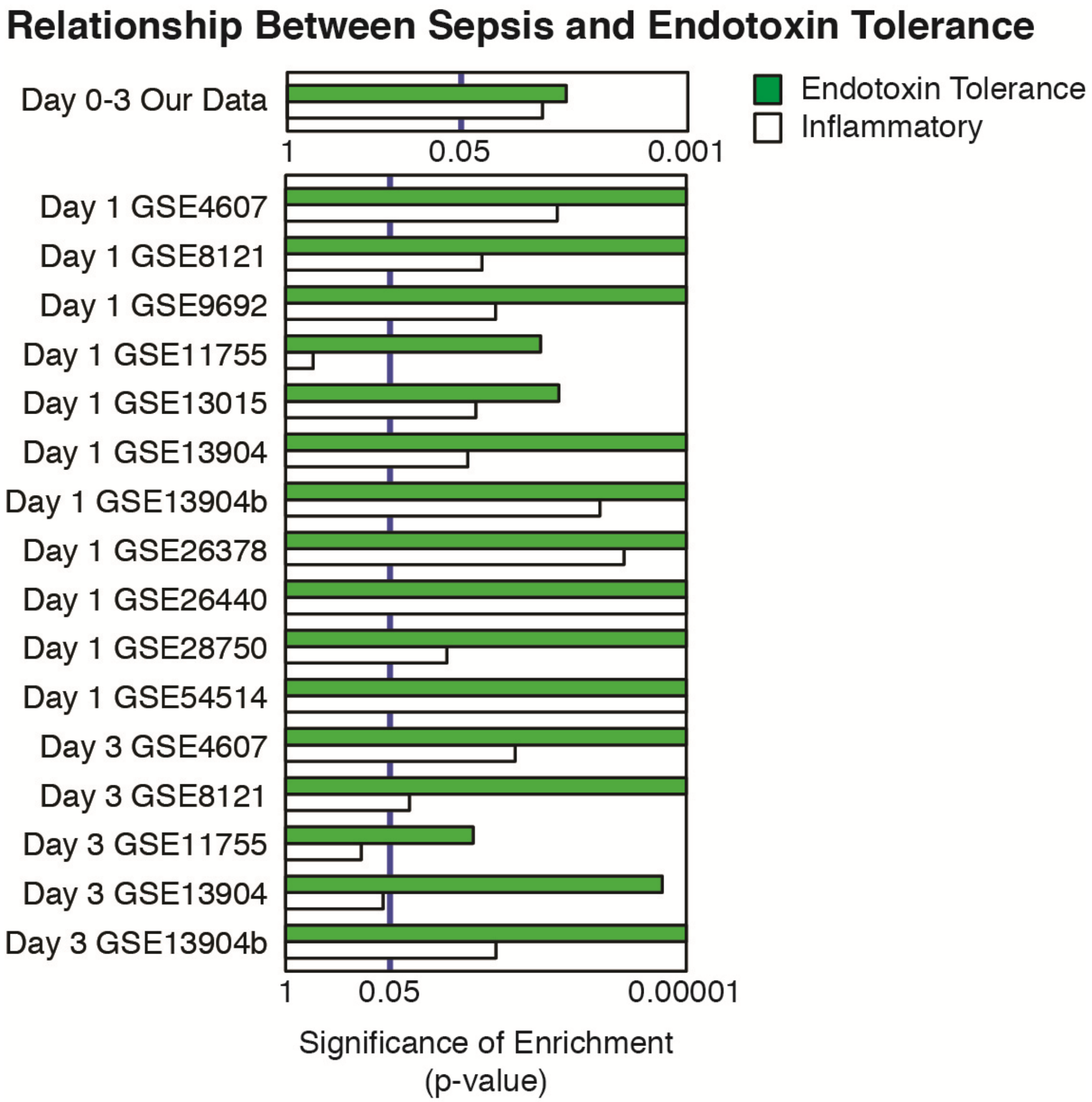

Supplementary Figure 2: Sepsis patients from published datasets generally showed a less significant association with the Inflammatory Signature. A gene-set test approach, ROAST, was used to characterize the enrichment of Inflammatory signature (white) relative to the Endotoxin Tolerance Signature (green) in sepsis patients cf. controls in 9 previously published datasets. All datasets contained sepsis patients recruited at days 1 and/or 3 post-ICU admission and were compared to 'healthy' controls. The ROAST gene-set test[14] was run with 99999 rotations so the most significant p-value resulting from this test is 0.00001. P-values from the ROAST gene-set test were graphed as log (1 / p-value), but the untransformed p-values are shown for ease of visualization.

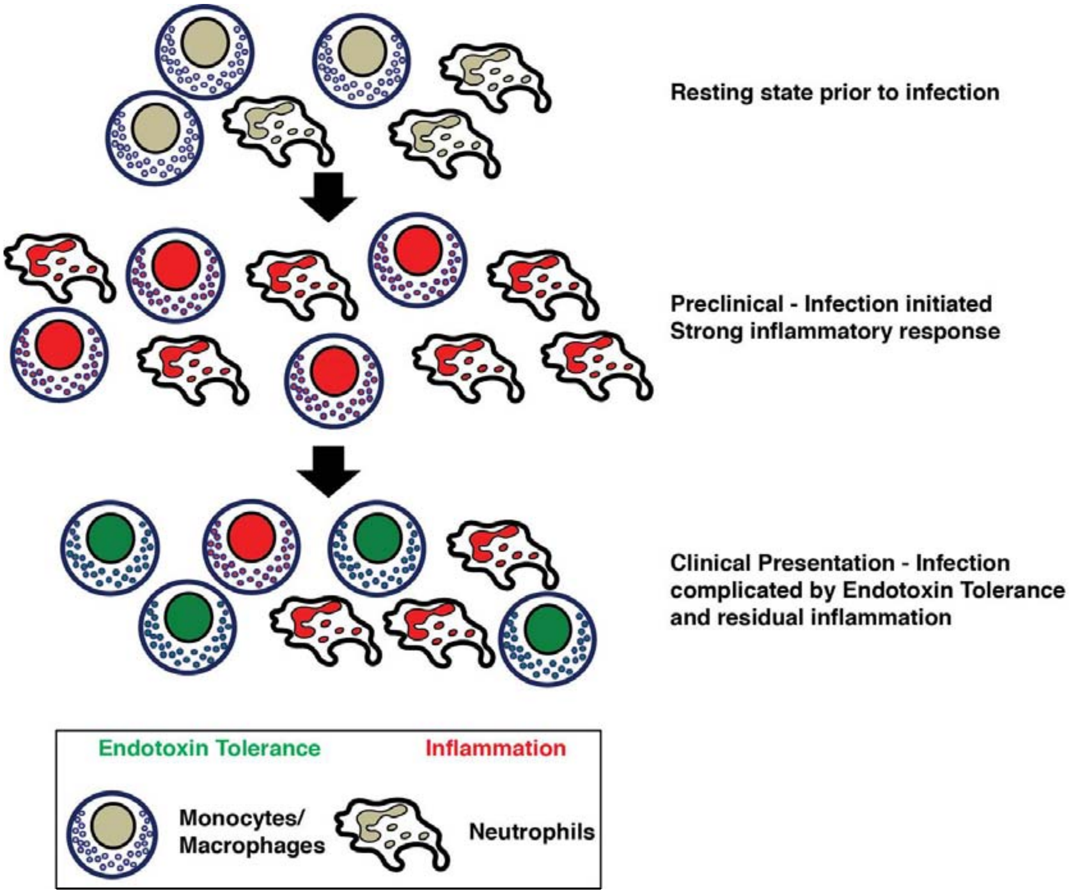

Supplementary Figure 3: Proposed model of the development of endotoxin tolerance during sepsis. Based on the findings here, we suggest that there is an initial (uncontrolled) pre-clinical infection, during which resting immune cells (grey) such as neutrophils, and monocytes/macrophages get activated (cells in red) resulting in the patient developing the first strong clinical symptoms. In septic patients it is proposed that a second endotoxin stimulus leads to the rapid activation of an endotoxin tolerance profile (cells in green). By the time of first hospital admission, this endotoxin tolerance profile predominates in PBMC systemically, while residual neutrophilic inflammation still occurs in this rapidly turning over population.

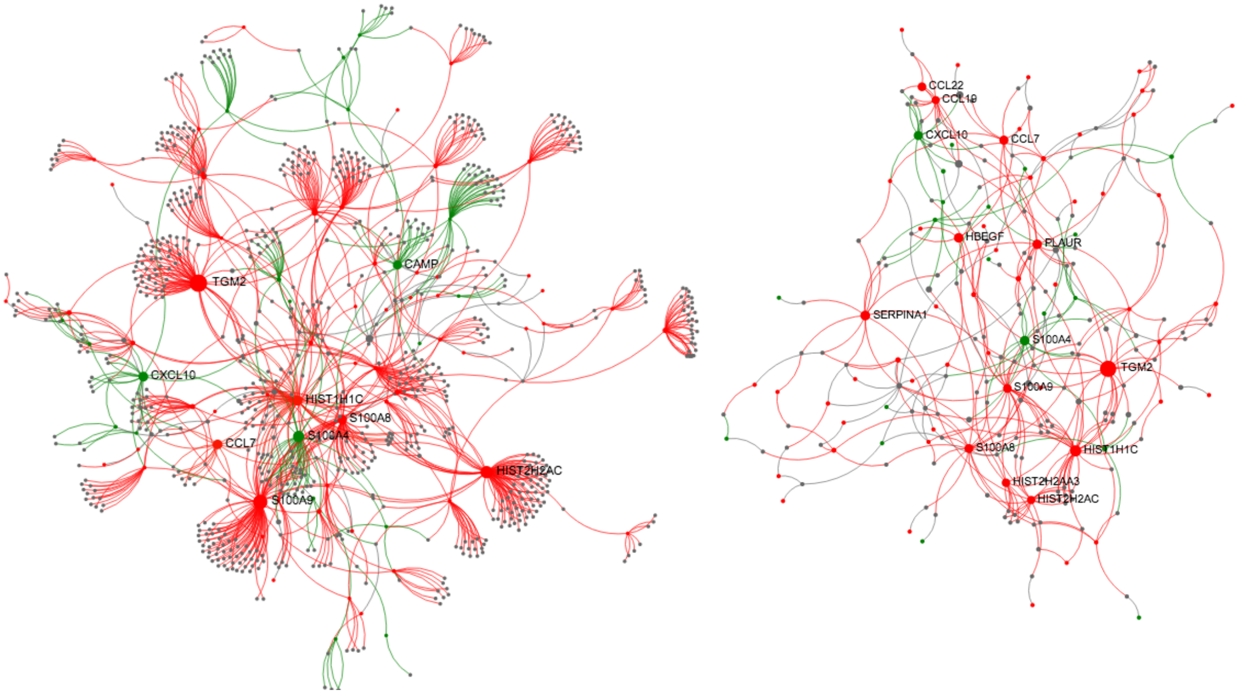

Supplementary Figure 4: Sub-network of genes from the Endotoxin Tolerance Signature created using a new Network drawing tool Network Analyst [ref. 35 of the main paper]. The fact that many of the 99 genes in the signature were tightly interconnected in the human cell implicates a biologically meaningful relationship between these genes; i.e. that these genes are co-regulated or are involved in a common purpose in the cell. The network was created by including first level interactors of the genes listed in Supplementary Table 1 and then subjected to analysis. Red nodes (genes) are upregulated, green down regulated and grey nodes are direct interactors of the dysregulated genes, lines represent "edges" and indicate experimentally proven interactions from InnateDB (www.innatedb.ca). Evident within the network are hub proteins with degree of cross talk with other nodes indicated by the size of the circles. In the right hand figure the entire Network is shown. In the left hand figure the central part of this network is made larger and the outermost interconnections removed to enable more detail to be observed.

SUPPLEMENTARY TABLES

Supplementary Table 1: Endotoxin Tolerance Signature genes and their relative expression in endotoxin tolerant PBMCs versus controls[12].

Endotoxin Tolerance Signature Genes		
**Gene Symbol**	**Description**	**Fold Change**
MT1G	Metallothionein 1G	61.1
MT1H	Metallothionein 1H	51.1
MT1M	Metallothionein 1M	23.8
CCL7	Chemokine (C-C motif) ligand 7	21.0
CCL24	Chemokine (C-C motif) ligand 24; Eotaxin-2	19.8
MT1F	Metallothionein 1F	16.2
MT1X	Metallothionein 1X	14.8
LILRA3	Leukocyte immunoglobulin-like receptor, subfamily A (without TM domain), member 3	14.0
C19orf59	Chromosome 19 open reading frame 59	12.6
MMP7	Matrix metallopeptidase 7 (matrilysin, uterine)	12.0
CA12	Carbonic anhydrase XII	8.2
CCL1	Chemokine (C-C motif) ligand 1; SCYA1	7.1
CCL22	Chemokine (C-C motif) ligand 22; MDC	7.0
PPBP	Pro-platelet basic protein (chemokine (C-X-C motif) ligand 7)	6.8
FPR1	Formyl peptide receptor 1	5.7
SERPINA1	Serpin peptidase inhibitor, Clade A (α-1 anti-proteinase, anti-trypsin), member 1	5.7
VCAN	Versican	5.3
FPR2	Formyl peptide receptor 2	4.9
CD93	CD93 molecule	4.6
RETN	Resistin	4.4
SERPINB7	Serpin peptidase inhibitor, Clade B (ovalbumin), member 7	4.3
IL3RA	Interleukin 3 receptor, alpha (low affinity)	4.2
ANKRD1	Ankyrin repeat domain 1 (cardiac muscle)	4.1
CCL19	Chemokine (C-C motif) ligand 19; MIP3β	4.1
HSD11B1	Hydroxysteroid (11-beta) dehydrogenase 1	4.1
KIAA1199	KIAA1199	4.1
HIST2H2AA3	Histone cluster 2, H2AA3	4.0
MARCO	Macrophage receptor with collagenous structure	3.7
S100A12	S100 calcium binding protein A12	3.7
HIST2H2AC	Histone cluster 2, H2AC	3.6
TREM1	Triggering receptor expressed on myeloid cells 1	3.5
PTGES	Prostaglandin E synthase	3.3
FBP1	Fructose-1,6-bisphosphatase 1	3.2
PDLIM7	PDZ and LIM domain 7 (enigma)	3.1

ADAMDEC1	ADAM-like, decysin 1	3.0
CYP27B1	Cytochrome P450, family 27, subfamily B, polypeptide 1	3.0
FCER2	Fc fragment of Ige, low affinity II, receptor for (CD23)	2.9
RHBDD2	Rhomboid domain containing 2	2.9
SLC16A10	Solute carrier family 16, member 10 (aromatic amino acid transporter)	2.9
MGST1	Microsomal glutathione S-transferase 1	2.7
PANX2	Pannexin 2	2.7
PLAUR	Plasminogen activator, Urokinase receptor	2.7
DPYSL3	Dihydropyrimidinase-like 3	2.6
LILRA5	Leukocyte immunoglobulin-like receptor, subfamily A (with TM domain), member 5	2.6
PTGR1	Prostaglandin reductase 1	2.6
CD14	CD14 molecule	2.5
HBEGF	Heparin-binding EGF-like growth factor	2.5
OLIG2	Oligodendrocyte lineage transcription factor 2	2.5
S100A9	S100 calcium binding protein A9	2.5
EMR3	EGF-like module containing, mucin-like, hormone receptor-like 3	2.4
HK2	Hexokinase 2	2.4
HPSE	Heparanase	2.4
GK	Glycerol kinase	2.3
HIST1H1C	Histone cluster 1, H1C	2.3
RAB13	RAB13, member RAS oncogene family	2.3
SLC7A11	Solute carrier family 7 (anionic amino acid transporter light chain, xc-system), member 11	2.3
CDK5RAP2	CDK5 regulatory subunit associated protein 2	2.2
DDIT4	DNA-damage-inducible transcript 4	2.2
GPR137B	G protein-coupled receptor 137B	2.2
NRIP3	Nuclear receptor interacting protein 3	2.2
CD300LF	CD300 molecule-like family member F	2.1
CYP1B1	Cytochrome P450, family 1, subfamily B, polypeptide 1	2.1
EMR1	EGF-like module containing, mucin-like, hormone receptor-like 1	2.1
HK3	Hexokinase 3 (white cell)	2.1
ITGB8	Integrin, beta 8	2.1
MYADM	Myeloid-associated differentiation marker	2.1
NEFH	Neurofilament, heavy polypeptide	2.1
S100A8	S100 calcium binding protein A8	2.1
TGM2	Transglutaminase 2	2.1
TMEM158	Transmembrane protein 158 (gene/pseudogene)	2.1
UPP1	Uridine phosphorylase 1	2.1
EGR2	Early growth response 2	2.0
FCER1G	Fc fragment of IgE, high affinity I, receptor for; gamma polypeptide	2.0
MXD1	MAX dimerization protein 1	2.0

PAPLN	Papilin, proteoglycan-like sulfated glycoprotein	2.0
PROCR	Protein C receptor, endothelial	2.0
ALCAM	Activated leukocyte cell adhesion molecule	-2.0
ADAM15	ADAM metallopeptidase domain 15	-2.1
PSTPIP2	Proline-serine-threonine phosphatase interacting protein 2	-2.1
TLR7	Toll-like receptor 7	-2.2
NQO1	NAD(P)H dehydrogenase, Quinone 1	-2.3
CTSK	Cathepsin K	-2.4
TSPAN4	Tetraspanin 4	-2.4
CST6	Cystatin E/M	-2.5
LY86	Lymphocyte antigen 86	-2.6
S100A4	S100 calcium binding protein A4	-2.7
PLD3	Phospholipase D family, member 3	-3.1
HTRA1	HTRA serine peptidase 1	-3.3
IL18BP	Interleukin 18 binding protein	-3.5
CPVL	Carboxypeptidase, Vitellogenic-like	-3.6
ALDH1A1	Aldehyde dehydrogenase 1 family, member A1	-3.8
RARRES1	Retinoic acid receptor responder (Tazarotene induced) 1	-3.8
CAMP	Cathelicidin antimicrobial peptide	-3.9
CST3	Cystatin C	-4.2
LIPA	Lipase A, lysosomal acid, cholesterol esterase	-4.5
DHRS9	Dehydrogenase/reductase (SDR family) member 9	-5.7
GPNMB	Glycoprotein (transmembrane) NMB	-8.1
CXCL10	Chemokine (C-X-C motif) ligand 10	-9.9
RNASE1	Ribonuclease, RNAse A family, 1 (pancreatic)	-10.4

Supplementary Table 2: The Inflammatory Signature genes and their relative expression in inflammatory PBMCs versus controls[12].

Inflammatory Signature Genes		
Symbol	Description	Fold Change
CCL20	Chemokine (C-C motif) ligand 20; MIP3α	14.6
CCL3L1	Chemokine (C-C motif) ligand 3-like 1; MIP1AP	9.2
G0S2	G0/G1switch 2	7.2
CFB	Complement factor B	6.1
AK4	Adenylate kinase 4	5.4
IFIT3	Interferon-induced protein with tetratricopeptide repeats 3	5.1
HERC5	HECT and RLD domain containing E3 ubiquitin protein ligase 5	4.8
PDSS1	Prenyl (decaprenyl) diphosphate synthase, Subunit 1	4.8
BATF	Basic leucine zipper transcription factor, ATF-like	4.7
DNAAF1	Dynein, axonemal, assembly factor 1	4.7
XAF1	XIAP associated factor 1	4.4
PIM2	PIM-2 oncogene	4.2
IFI44	Interferon-induced protein 44	3.7
F3	Coagulation factor III (thromboplastin, tissue factor)	3.6
FAM129A	Family with sequence similarity 129, member A	3.5
IFIT2	Interferon-induced protein with tetratricopeptide repeats 2	3.4
KCNJ2	Potassium inwardly-rectifying channel, subfamily J, member 2	3.4
MX2	Myxovirus (influenza virus) resistance 2 (mouse)	3.4
EIF2AK2	Eukaryotic translation initiation factor 2-alpha kinase 2	3.2
CCL3L3	Chemokine (C-C motif) ligand 3-like 3; LD78	3.1
IRF7	Interferon regulatory factor 7	3.1
CXCL2	Chemokine (C-X-C motif) ligand 2; MIP2α	3.0
FFAR2	Free fatty acid receptor 2	3.0
RIPK2	Receptor-interacting serine-threonine kinase 2	3.0
ADORA2A	Adenosine A2a receptor	2.9
SAMD9L	Sterile alpha motif domain containing 9-like	2.9
GRAMD1A	GRAM domain containing 1A	2.8
SOD2	Superoxide dismutase 2, mitochondrial	2.8
SOCS1	Suppressor of cytokine signaling 1	2.7
CD80	CD80 molecule	2.6
TNF	Tumor necrosis factor	2.6
CASP5	Caspase 5, apoptosis-related cysteine peptidase	2.5
CD83	CD83 molecule	2.5
IFI35	Interferon-induced protein 35	2.5
PIM1	Pim-1 oncogene	2.5
SLAMF7	SLAM family member 7	2.5

TRIM25	Tripartite motif containing 25	2.5
C1orf122	Chromosome 1 open reading frame 122	2.4
GBP4	Guanylate binding protein 4	2.4
PIM3	Pim-3 oncogene	2.4
GBP2	Guanylate binding protein 2, interferon-inducible	2.3
RNF144B	Ring finger protein 144B	2.3
TXN	Thioredoxin	2.3
YRDC	Yrdc domain containing (E. Coli)	2.3
ALCAM	Activated leukocyte cell adhesion molecule	2.2
ANTXR2	Anthrax toxin receptor 2	2.2
ISG20	Interferon stimulated exonuclease gene 20kda	2.2
OASL	2'-5'-oligoadenylate synthetase-like	2.2
PARP9	Poly (ADP-ribose) polymerase family, member 9	2.2
PTX3	Pentraxin 3, long	2.2
TNFAIP2	Tumor necrosis factor, alpha-induced protein 2	2.2
TNFSF10	Tumor necrosis factor (ligand) superfamily, member 10	2.2
B4GALT5	UDP-Gal:betaglcnac beta 1,4- galactosyltransferase, polypeptide 5	2.1
BCL3	B-cell CLL/lymphoma 3	2.1
EDN1	Endothelin 1	2.1
GADD45B	Growth arrest and DNA-damage-inducible, beta	2.1
IRAK2	Interleukin-1 receptor-associated kinase 2	2.1
JUNB	Jun B proto-oncogene	2.1
MTF1	Metal-regulatory transcription factor 1	2.1
NFKB2	Nuclear factor of kappa light polypeptide gene enhancer in B-cells 2 (p49/p100)	2.1
SAMD9	Sterile alpha motif domain containing 9	2.1
UPB1	Ureidopropionase, beta	2.1
GCH1	GTP cyclohydrolase 1	2.0
HSH2D	Hematopoietic SH2 domain containing	2.0
NFKBIZ	Nuclear factor of kappa light polypeptide gene enhancer in B-cells inhibitor, zeta	2.0
TNIP1	TNFAIP3 interacting protein 1	2.0
ZC3H12A	Zinc finger CCCH-type containing 12A	2.0
CORO1B	Coronin, actin binding protein, 1B	-2.0
H2AFY	H2A histone family, member Y	-2.0
IFFO1	Intermediate filament family orphan 1	-2.0
SPIRE1	Spire homolog 1 (Drosophila)	-2.0
TSC22D3	TSC22 domain family, member 3	-2.0
CSF1R	Colony stimulating factor 1 receptor	-2.1
PLIN2	Perilipin 2	-2.1
ZMIZ1	Zinc finger, MIZ-type containing 1	-2.1
CTSB	Cathepsin B	-2.2

LPAR6	Lysophosphatidic acid receptor 6	-2.2
MS4A7	Membrane-spanning 4-domains, subfamily A, member 7	-2.2
SLAMF8	SLAM family member 8	-2.2
IDH1	Isocitrate dehydrogenase 1 (NADP+), soluble	-2.3
LTA4H	Leukotriene A4 hydrolase	-2.4
CAMK1	Calcium/calmodulin-dependent protein kinase I	-2.5
CORO1C	Coronin, actin binding protein, 1C	-2.5
CLEC10A	C-type lectin domain family 10, member A	-2.9
CD86	CD86 molecule	-3.1
PDK4	Pyruvate dehydrogenase kinase, isozyme 4	-3.1
ACP5	Acid phosphatase 5, tartrate resistant	-3.2
HAVCR2	Hepatitis A virus cellular receptor 2	-3.2
ASGR1	Asialoglycoprotein receptor 1	-3.4
NCEH1	Neutral cholesterol ester hydrolase 1	-3.6
RCBTB2	Regulator of chromosome condensation (RCC1) and BTB (POZ) domain containing protein 2	-3.9
ADAP2	ArfGAP with dual PH domains 2	-4.6
HMOX1	Heme oxygenase (decycling) 1	-5.5

Supplementary Table 3: Description of analyzed public Sepsis datasets. Microarray data were downloaded from the repository Gene Expression Omnibus (GEO); http://www.bioconductor.org/packages/2.12/bioc/html/GEOquery.html. The associated papers (given by Pubmed number), numbers of patients and controls analyzed and details of the specific studies are presented (inclusion criteria as per footnote #). The Study description is included as per the footnote*. Array Platform was A. GPL570 [HG-U133_Plus_2] Affymetrix Human Genome U133 Plus 2.0 Array; B. GPL6947 Illumina HumanHT-12 V3.0 expression beadchip. R = RNA-Seq.

N*	GEO ID	Samples selected here	Samples (Sepsis/ Controls)	Location; Cell Type; Time of sample collection	Pubmed #; Year	Array Platform
1	GSE 28750	Used only samples from sepsis subjects and healthy controls. Post-surgery subject group were excluded from the analysis.	10/ 20	Australia; Leukocytes; ICU <24H>	21682927; 2011	A
2	GSE 13015	Used only samples with sepsis due to organisms other than B. pseudomallei, Controls were healthy without comorbidities.	24/ 3	Thailand; Whole blood; Within 24h of sepsis diagnosis	19903332; 2009	B
3	GSE 9692	Used all samples provided by the study.	30/ 15	USA; Leukocytes; ICU <24H>	18460642: 2007	A
4	GSE 26378	Used all samples provided by the study.	82/ 21	USA; Leukocytes; ICU <24H>	21738952; 2011	A
5	GSE 26440	Used all samples provided by the study.	98/ 32	USA; Leukocytes; ICU <24H>	21738952; 2011	A
6	GSE 4607	Used only samples from subjects undergoing septic shock collected at day 1 and day 3 post-ICU admission. SIRS & SIRS-resolved subject samples were excluded.	69/ 15	USA; Leukocytes; ICU <24H>	17374846; 2006	A
7	GSE 8121	Used all samples provided by the study.	60/ 15	USA; Leukocytes; ICU <24H>	17932561; 2007	A
8	GSE 11755	Used only samples from septic subjects after 24 hours (day 1) and 72 hours (day 3) of ICU admission. Samples taken at 8 hours post-admission were excluded from the analysis.	5/ 3	Netherlands; Leukocytes; ICU <24H>	23842590; 2008	A
9	GSE 13904	Used only Samples from sepsis and septic shock subjects. SIRS subjects were excluded from analysis.	158/ 18	USA Leukocytes; ICU <24H>	19325468; 2008	A
10	GSE 54514	Used only Day 1 sepsis samples to avoid bias from the patients lost to follow up in Day 2-5. Used Day 5 controls due to high quality	35/18	Australia; Leukocytes; ICU <24H>	23807251; 2013	B

Inclusion criteria utilized in selecting studies were: 1) Inclusion of Sepsis or Septic Shock patients; 2) Cross-sectional or longitudinal cohort studies; (3) Use of whole blood or purified leukocyte populations; 4) Inclusion of paediatric or adult patients; 5) Inclusion of healthy subjects used as controls; 6) Only datasets that passed the bioinformatics tests (normalization and integrity check) for sample quality; and 7) Only datasets published as part of a study in a scientific journal.

*** Study Design**

1. GSE 28750. Cross-sectional. Multi-centre, prospective clinical trial conducted across 4 tertiary critical care settings in Australia. Sepsis patients were recruited if they met the 1992 Consensus Statement criteria and had clinical evidence of systemic infection based on microbiology diagnoses. Healthy subjects were used as normal controls in the study.

2. GSE 13015. Cross-sectional. Study of patients with sepsis with a positive blood culture due to *Burkholderia pseudomallei*, and sepsis due to other organisms cf. non-infected controls

3. GSE 9692. Cross-sectional. Children <10 yr of age admitted to the pediatric intensive care unit (PICU), with pediatric-specific criteria for septic shock. Normal control patients were recruited from the participating institutions using the following exclusion criteria: a recent febrile illness (within 2 wk), recent use of anti-inflammatory medications (within 2 wk), or any history of chronic or acute disease associated with inflammation.

4. GSE 26378. Cross-sectional. Expression data from children with septic shock was generated using whole blood-derived RNA samples representing the first 24 hours of admission to the PICU. Healthy subjects (children) were used as normal controls in the study.

5. GSE 26440. Cross-sectional. Expression data from children with septic shock were generated using whole blood-derived RNA samples representing the first 24 hours of admission to the PICU. Healthy subjects (children) were used as normal controls in the study.

6. GSE 4607. Longitudinal. Children < 10 years of age admitted to the pediatric intensive care unit and meeting the criteria for either SIRS or septic shock were eligible for the study. Control patients were recruited from the outpatient or inpatient departments of the participating institutions using the following exclusion criteria: a recent febrile illness (within 2 weeks), recent use of anti-inflammatory medications (within 2 weeks), or any history of chronic or acute disease associated with inflammation.

7. GSE 8121. Longitudinal. Genome-level expression profiles were generated from whole blood-derived RNA of children with septic shock corresponding to day 1 and day 3 of septic shock, respectively. Control patients were recruited from the participating institutions using the following exclusion criteria: a recent febrile illness (within two weeks), recent use of anti-inflammatory medications (within two weeks), or any history of chronic or acute disease associated with inflammation.

8. GSE 11755. Longitudinal. Prospective case-control study, six children with meningococcal sepsis were included. Blood was drawn at four time points (t=0, t=8, t=24 and t=72 h after admission to the pediatric intensive care unit. Healthy subjects (children) were used as normal controls in the study.

9. GSE 13904. Longitudinal. Genome-level expression profiles of critically ill children representing the systemic inflammatory response syndrome (SIRS), sepsis, and septic shock

spectrum at day 1 and day 3 post-admission. Healthy subjects (children) were used as normal controls in the study.

10. GSE 54514. Longitudinal. Genome Level expression profile of critically ill adults including sepsis survivors (n=26), sepsis non-survivors (n=9), and healthy controls (n=18).

Supplementary Table 4: Details of individual patients recruited in this study. NB all patients were suspected to be infected based on the physician requesting microbial cultures.

**Lab ID**	**ICU admission or not**[1]	**Number organ failures (within 48 hr of suspected sepsis)**	**Diagnostic Criteria**[2]						
			**Confirmed Microbiology (most within 48 hr of suspected sepsis)**	Triage blood pressure systolic/ diastolic	**Initial WBC**	**Triage Temp. °C**	**Triage Heart Rate**	Triage Respir-atory Rate	First Partial CO_2
**SEPSIS GROUP**									
612920	ICU	4	Positive	94/62	**17**	37.8	**170**	**40**	NA[3]
154114	ICU	3	Positive	73/45	**22.4**	36.7	73	**32**	38
297580	ICU	4	Positive	86/40	10.4	**35.3**	56	16	**30**
212463	ICU	3	Positive	165/101	**19.1**	37.5	**98**	**34**	71
708631	ICU	2	Positive	100/61	8	**38.2**	**96**	10	42
799587	ICU	5	Positive	84/45	7.2	37	**127**	**22**	**25**
795380	ICU	3	Positive	139/90	4.2	**30.4**	53	**22**	61
913994	ICU	4	Positive	81/62	10.4	**39.3**	**139**	**40**	26
889485	ICU	4	Positive	67/53	**16.3**	36.1	**142**	**26**	**30**
137731	ICU	3	Positive	130/78	**19.8**	36.4	**100**	16	52
862476	ICU	4	Positive	136/80	**25.8**	**39.2**	**110**	**30**	49
864637	ICU	4	Positive	83/54	11.3	37.2	**126**	**26**	51
980414	ICU	3	Positive	112/62	**26.3**	37.9	**126**	**44**	35
375523	ICU	4	Positive	134/58	**37.7**	**38.5**	**133**	**34**	NA
364132	Non-ICU	0	Positive	98/68	9.4	**38.3**	**119**	**22**	NA
450578	Non-ICU	0	Positive	86/44	**18.4**	37.7	86	18	NA
694402	Non-ICU	1	Positive	175/81	**2.2**	37.4	**118**	**22**	NA
732740	Non-ICU	2	Positive	155/83	**3.2**	37.1	**107**	**22**	NA
826967	Non-ICU	1	Positive	129/66	**23.8**	37.2	**109**	20	NA
300271	ICU	3	Negative[6]	103/57	**15.3**	36.7	**102**	**22**	42
679797	ICU	3	Negative	96/57	**40**	36.9	**127**	**24**	NA
266144	ICU	4	Negative	217/121	**2.1**	36.5	**135**	20	**28**
602395	ICU	3	Negative	105/95	**12.9**	37.5	**92**	NA	37
476146	ICU	3	Negative	106	**15.2**	36.2	**105**	-[4]	44
853176	Non ICU	0	Negative	139/69	**14.4**	37.1	**105**	**23**	NA
220171	Non ICU	2	Negative	76/51	**3.3**	**39**	**109**	18	NA
581691	Non ICU	1	Negative	102/57	**19.5**	36.7	**105**	**24**	NA
823914	Non ICU	0	Negative	90/52	**12.3**	37.2	**138**	**28**	NA
155286	Non ICU	0	Negative	141/79	**14.8**	36.4	**114**	20	NA

658301	Non ICU	1	Negative	114/76	**16.6**	36.7	**119**	16	NA
800267	Non ICU	1	Negative	143/97	6.3	36.5	**130**	**24**	NA
235545	Non ICU	0	Negative	103/78	**21.8**	36.4	**105**	18	NA
342306	Non ICU	1	Negative	120/73	**12.1**	36.7	**136**	16	NA
468026	Non ICU	0	Negative	171/110	6.4	37.3	**101**	**22**	NA
522087	Non ICU	0	Negative	124/62	7	36.7	**120**	**38**	NA
716574	Non ICU	1	Negative	117/89	5.8	36.4	**106**	**40**	NA
746024	Non ICU	1	Negative	91/55	**19.1**	37.4	90	**28**	NA
**NO SEPSIS GROUP**									
402569	ICU	3	Positive	123/74	10.4	37.1	74	16	**31**
941715	Non ICU	0	Positive	119/65	5.1	36.8	82	16	NA
583654	Non ICU	0	Positive	180/96	7.6	36.4	64	20	NA
237093	Non ICU	0	Positive	102/58	10.3	36.6	82	16	NA
355472	Non ICU	1	Positive	147/70	**13.4**	38	66	20	NA
416442	Non ICU	2	Positive	133/62	7	**39.5**	89	20	NA
439362	Non ICU	1	Positive	146/84	5.6	36.8	89	16	NA
701198	Non ICU	1	Positive	147/68	5.6	36.4	**107**	18	NA
583577	ICU	2	Negative	203/111	11.2	36	85	**32**	73
749752	ICU	3	Negative	85/50	**12.3**	36.6	60	18	NA
673143	ICU	4	Negative	162/87	9.1	36.8	90	20	40
362763	ICU	3	Negative	126/60	7.3	37	90	-[3]	39
377121	ICU	1	Negative	128/88	4.8	37.2	**115**	20	36
288187	Non-ICU	0	Negative	95/57	9.7	36.9	**98**	20	NA
993234	Non-ICU	0	Negative	152/57	6.8	36.6	88	**24**	NA
890426	Non-ICU	1	Negative	135/75	8.1	37.6	**105**	16	NA
290697	Non-ICU	0	Negative	100/61	8.4	36.7	**91**	20	NA
104582	Non-ICU	0	Negative	140/88	**17.1**	36.6	74	20	NA
245286	Non-ICU	0	Negative	136/75	5.7	36.8	**105**	20	NA
417642	Non-ICU	0	Negative	120/60	6.9	36.7	77	**24**	NA
911536	Non-ICU	0	Negative	123/80	4.3	39.4	90	18	NA
346081	Non-ICU	0	Negative	167/70	**2.1**	36.9	72	20	NA
449469	Non-ICU	0	Negative	127/78	5.9	37.3	**104**	16	NA
568243	Non-ICU	1	Negative	159/98	6.7	36.9	**93**	18	NA
695232	Non-ICU	1	Negative	142/84	**19.1**	37.8	90	16	NA
770905	Non-ICU	0	Negative	123/60	**13.2**	35.4	73	16	NA
929438	Non-ICU	1	Negative	170/102	**12.8**	37.4	83	16	NA

602005	Non-ICU	0	None[5]	130/66	**1.8**	36.6	64	14	NA
145305	Non-ICU	0	Negative	142/75	8.4	36.8	67	18	NA
366713	Non-ICU	0	Negative	91/58	10.8	36.6	71	16	NA
332278	Non-ICU	1	Negative	99/59	8.5	36.6	86	16	NA
379752	Non-ICU	0	Negative	130/69	10.6	36.9	65	16	NA
669339	Non-ICU	0	Negative	123/80	5.9	37	77	16	NA
310017	Non-ICU	0	Negative	141/96	11.4	36.9	88	20	NA
504886	Non-ICU	0	Negative	99/55	NA	36.4	66	16	NA

[1]Indicates whether the patient was transferred to the ICU after first clinical examination
[2]Diagnostic Criteria for Sepsis as per references 3 and 22-24 of the main paper; Blood Pressure, Respiratory Rate and Partial CO_2 are no longer criteria but were added as additional information.
[3]NA: indicates not available
[4]Patient was ventilated
[5]None means no culture was requested.
[6]While culture was negative, patients were originally considered to be possibly infected since the attending physician ordered one or more cultures to be performed.

Supplementary Table 5. Statistics regarding Organ Dysfunction and Sites of Infection. A. New organ dysfunction was defined by the presence of cardiovascular dysfunction (treatment with a vasopressor (norepinephrine, epinephrine, phenylephrine), acute respiratory distress syndrome (bilateral pulmonary infiltrates, need for mechanical ventilation and PaO2/FiO2 < 300 mmHg), coagulopathy (platelet count <80/µL), hepatic dysfunction (bilirubin >34 µmol/L) and acute kidney injury (a serum creatinine rise ≥26.5µmol/L or ≥1.5 fold from baseline. NB. No patients were observed with sepsis-associated encephalopathy. B. Multiple sites were sampled (as per the first column in part B) and the infections identified at each site described.

**A. Site of Organ Dysfunction**	**Number of Patients**
Lung (Respiratory Failure)	22
Kidney (Acute Kidney Injury)	41
Liver	9
Cardiovascular System	22
Blood (Coagulation)	9
**B. Site of Infections**	
Blood	9
Urinary Tract	10
Respiratory Tract	6
Gastrointestinal Tract	4
Skin and Soft Tissues	3
Bone	1
